# A comprehensive review of finerenone—a third-generation non-steroidal mineralocorticoid receptor antagonist

**DOI:** 10.3389/fcvm.2024.1476029

**Published:** 2024-09-23

**Authors:** Shuhui Zhai, Baisheng Ma, Weiwei Chen, Qini Zhao

**Affiliations:** Department of Cardiology, China-Japan Union Hospital of Jilin University, Changchun, China

**Keywords:** aldosterone, eplerenone, spironolactone, finerenone, mineralocorticoid receptor antagonists (MRA)

## Abstract

Multiple studies have shown that finerenone (BAY 94-8862), a third-generation non-steroidal mineralocorticoid receptor antagonist (MRA), possesses different or superior mechanisms of action to traditional MRAs. Specifically, animal and cell-based experiments have demonstrated that this compound exerts multiple effects including fibrosis inhibition, reduced pulmonary artery pressure, improved diabetic retinopathy, enhanced endothelial functions, metabolic optimization as well as reduced oxidative stress, thereby exerting overall positive effects on renal and cardiovascular diseases. Consequently, clinical research, such as the FIGARO-DKD and FIDELIO-DKD trials, has demonstrated dual benefits for patients with type 2 diabetes mellitus and chronic kidney disease (T2DM-CKD), especially by validating MRAs’ potential in reducing risks of renal and cardiovascular composite endpoints. Currently, cardiovascular indications for finerenone are limited to patients with T2DM-CKD, while its use in non-T2DM CKD patients remains at clinical trial stages. Despite showing good safety and efficacy in T2DM-CKD patients, there are insufficient corresponding data for those presenting chronic kidney disease without diabetes (ndCKD). Furthermore, the application of this compound in diseases such as primary aldosteronism and its association with cancer risk need to be further validated through larger-scale and longer-term clinical studies. Nevertheless, the development of finerenone provides an additional option for treating cardiovascular and renal diseases. With further research, it is expected that finerenone will be relevant to a broader range of CKD patient populations by addressing current knowledge gaps to comprehensively evaluate its clinical value and potentially alter existing treatment strategies. The current review aims to comprehensively analyze the basic research and clinical advancements involving finerenone in order to explore its prospects for treating cardiovascular and renal diseases, while addressing unmet needs in current treatment strategies. Additionally, through a comprehensive analysis of relevant research findings, a deeper understanding of finerenone's drug characteristics will be provided alongside scientific guidance for future treatment strategies and their clinical significance.

## 1 Introduction

A common mechanism underlying the pathophysiology of diseases, such as chronic kidney disease, hypertension and heart failure, is the activation of the renin-angiotensin-aldosterone system (RAAS) (1, 2). This resulted in the emergence of therapeutic strategies that target the RAAS system, with one example being the development of different mineralocorticoid receptor antagonists (MRA) such as the first-generation spironolactone, the second-generation eplerenone or the more recent third-generation finerenone (3). The clinical application of these MRAs and their protective effects on the cardiovascular system have been widely established (4–6), but in comparison with the older-generation ones, finerenone demonstrates greater selectivity and is associated with a lower risk of adverse reactions (7). Although spironolactone may impact glycemic control to a certain extent in some cases, there is currently no definitive evidence to suggest that finerenone worsens glycemic regulation (8, 9). Moreover, the distribution of finerenone between the kidneys and the heart is more balanced, thereby reducing the risk of significant renal accumulation of the drug (10).

In this article, the latest research developments on finerenone are comprehensively examined. In particular, its significant anti-cardiac fibrosis effects, its ability to prevent arrhythmias and improve endothelial functions as well as its potential in treating diabetic retinopathy and delaying diabetic nephropathy's progression are demonstrated in the light of preclinical studies (11–17). Additionally, finerenone can exert positive effects on metabolic functions, thereby opening new avenues for the treatment of metabolic disorders (18, 19). Altogether, these findings lay the groundwork for considering finerenone's application in clinical settings. Furthermore, key clinical trials, including FIGARO-DKD, FIDELITY, ARTS, FIDELIO-DKD and their sub-studies are reviewed alongside other randomized controlled trials (RCTs) that further validate finerenone's potential in treating cardio-renal diseases (20–25). In doing so, this article aims to identify unmet needs in current treatment strategies, comprehensively assess the opportunities and challenges of finerenone's application and provide a theoretical basis for future research in view of broadening its potential.

## 2 RAAS and MRA

The RAAS system is a crucial neuroendocrine regulatory mechanism which maintains the human body's homeostasis (26, 27). When fully activated, this system induces changes such as vasoconstriction as well as the retention of water and sodium (28). Furthermore, basic research has shown that its activation could be associated with oxidative stress, hypertrophy, fibrosis and inflammation, while its overactivation could lead to various pathophysiological changes, including alterations to renal tissues and cardiovascular diseases such as heart failure, atrial fibrillation, hypertension and vascular remodeling (1, 29–32). However, beneficial effects have been widely observed in the progression of cardiovascular diseases ever since spironolactone and eplerenone (the first- and second-generation MRAs, respectively) have been used (33, 34). Similarly, finerenone, the latest generation of MRA, has gradually been gaining attention as evidenced from the results of multiple clinical studies, the meta-analyses reported in several articles as well as the recommendations from relevant guidelines (5, 19, 22, 35, 36). Indeed, as a novel group of non-steroidal MRA, finerenone offers unique advantages in comparison with eplerenone or spironolactone: For instance, the two earlier generation compounds exhibit greater renal accumulation as opposed to cardiac accumulation, while finerenone is distributed more evenly between the two organs, thereby reducing risks of renal accumulation and subsequent cases of hyperkalemia (29, 37, 38). Moreover, through its non-steroidal chemical structure, finerenone effectively combines eplerenone's selectivity and spironolactone's efficacy to prevent structural and functional damage to the heart and kidneys (10, 29).

### 2.1 Steroidal MRAs vs. finerenone

**Figure 1:**
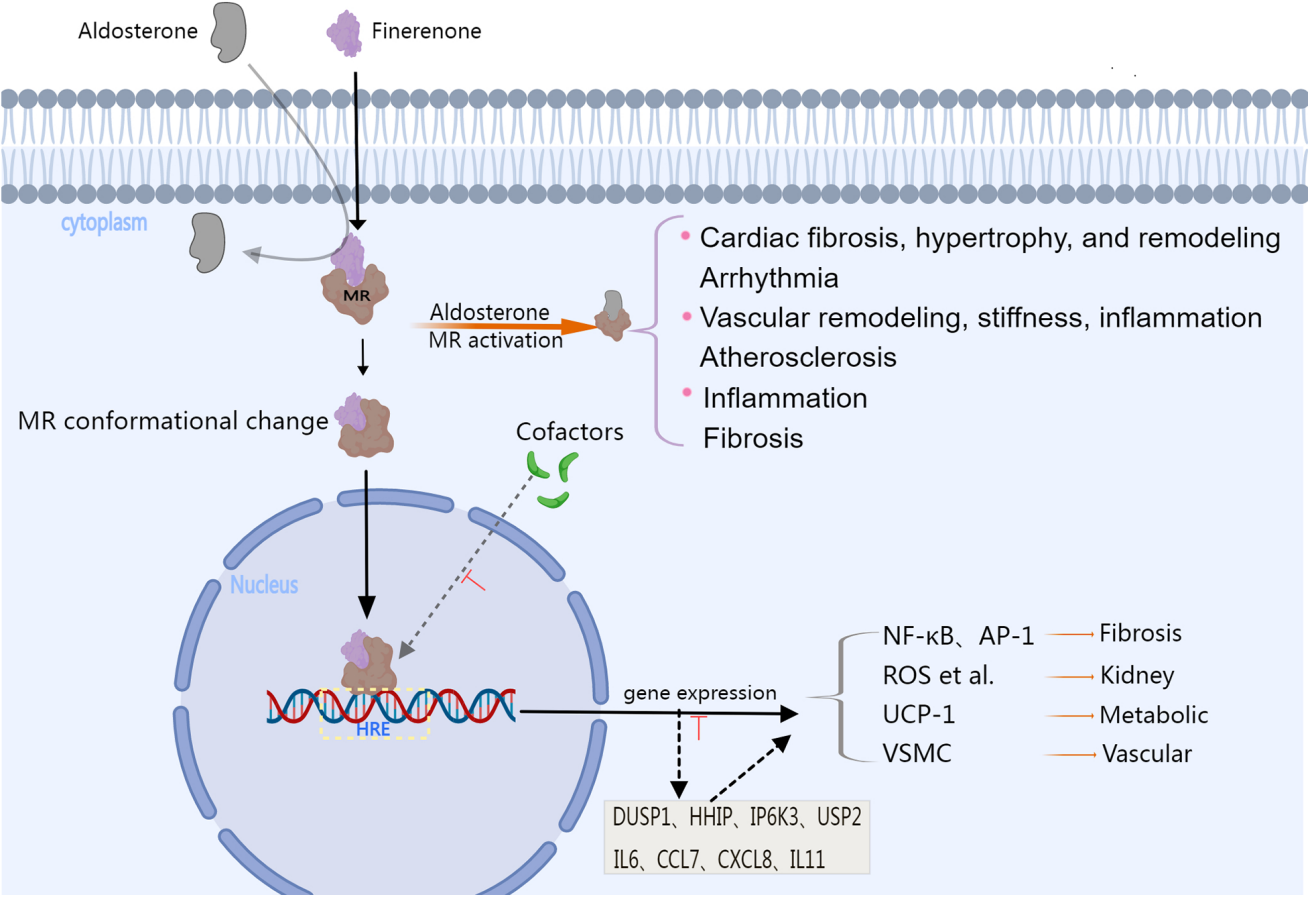
The image illustrates the physiological changes that occur after MR activation, as well as the pharmacological mechanisms and efficacy associated with finerenone. Aldosterone binds to the mineralocorticoid receptor (MR), causing a conformational change, and then it moves into the cell nucleus. Over-activation of the MR can promote the expression of genes that are pro-inflammatory and pro-fibrosis, as well as the activation of signaling pathways related to the progression of diseases such as heart and vascular disease. Steroid MRAs can also interact with co-factors that affect gene transcription, serving as partial MR agonists. Finerenone is a highly lipophilic non-steroidal mineralocorticoid receptor antagonist,it can inhibit the recruitment of cofactors from the cytoplasm to the nucleus of the MR, and this inhibition of the binding of MR co-factors can occur even in the absence of aldosterone. Moreover, the gene regulatory profile of finerenone is different from that of steroidal MRAs. Finerenone significantly suppresses the transcription of genes induced by aldosterone, including DUSP1, HHIP, IP6K3, and USP2, and is more effective in antagonizing the expression of inflammatory genes such as IL6, CCL7, CXCL8, and IL11 compared to spironolactone. Finerenone has stronger anti-inflammatory and antifibrotic activities than steroidal MRAs, providing stronger protection for the heart, kidneys, metabolism, and vascular disease. MR, mineralocorticoid receptor; HRE, hormone response element; NF-κB, nuclear factor kappa-light-chain-enhancer of activated B cell; AP-1, activator protein-1; ROS, reactive oxygen species; UCP-1, Uncoupling Protein 1; VSMC, Vascular Smooth Muscle Cell; DUSP1, dual specificity protein phosphatase 1; HHIP, hedgehog interacting protein; IP6K3, inositol hexakisphosphate kinase 3; USP2, ubiquitin specific peptidase 2; IL6, Interleukin 6; CCL7, C-C motif chemokine ligand 7; CXCL8, C-X-C motif chemokine ligand 8; IL11, Interleukin 11. Created with MedPeer.

As a novel selective MRA, finerenone (BAY 94-8862) binds to the mineralocorticoid receptor (MR) with stronger affinity compared with eplerenone and spironolactone (39). Furthermore, its pharmacological characteristics are also distinct from steroidal MRAs (6), with finerenone inhibiting MR signaling at multiple levels. For instance, when bound to MR, it inhibits conformational changes between aldosterone or other mineralocorticoids and MR. Additionally, it reduces MR accumulation and its turnover in the cell nucleus, thus inhibiting the recruitment of transcription co-factors which may occur even in the absence of aldosterone. This contrasts with the mechanism of steroidal MRAs which can only act as partial agonists during cofactor recruitment (40–42). In addition, spironolactone can exhibit potent efficacy but lacks selectivity, while eplerenone offers greater selectivity, albeit with lower potency (41). Therefore, overall, finerenone demonstrates superior selectivity for genes within the cell nucleus as well as better efficacy in aldosterone's inhibition compared with the steroidal MRAs (10, 38). This intricate process is visually depicted in Figure 1 and Table 1 presents key pharmacodynamic and pharmacokinetic characteristics of steroidal and finerenone (2, 29, 37, 40, 43–45).

**Table 1:** Steroidal MRAs vs. finerenone.

	Steroidal MRAs		Nonsteroidal
	Spironolactone	Eplerenone	Finerenone
Structural properties	Flat	Flat	Bulky
Potency to MR	+++	+	+++
Selectivity to MR	+	++	+++
Tissue distribution (in rodents)	Kidney > heart	Kidney > heart	Kidney = heart
Half-life	>20 h	4–6 h	2–3 h
Effect on BP	+++	++	+
Sexual side effects	+++	++	-
Hyperkalemia	+++	++	+

## 3 Finerenone's preclinical results

**Figure 2:**
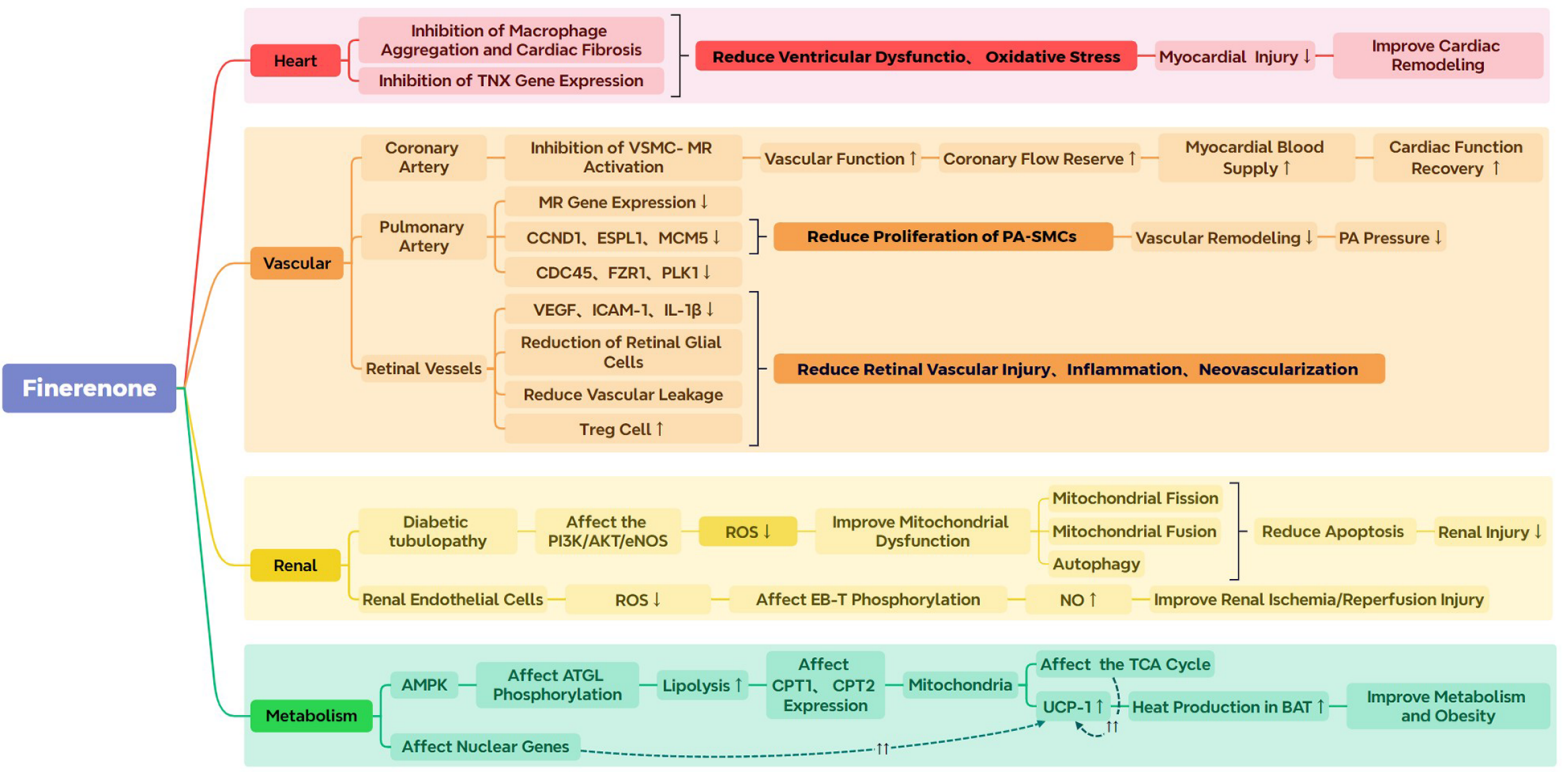
The image displays a summary of the preclinical research related to finerenone. PA, Pulmonary arterial; VSMC-MR, vascular smooth muscle cell- mineralocorticoid receptor. ROS, reactive oxygen species; CCND1, cyclin D1; ESPL1, extra spindle poles-like 1; MCM5, minichromosome maintenance 5; CDC45, cell division cycle 45; FZR1, fizzy/cell division cycle-associated protein 1; PLK1, polo-like kinase 1; VEGF, vascular endothelial growth factor; ICAM-1, intracellular adhesion molecule 1; IL-1β, interleukin-1β; EB-T, endothelin-B; AMPK, AMP-activated protein kinase; ATGL, adipose triglyceride lipase; UCP-1, Uncoupling Protein 1; TCA Cycle, tricarboxylic acid cycle; BAT, brown adipose tissue.

Based on the reported pharmacological mechanisms and existing clinical research on finerenone, its four main effects are summarized in the subsequent sections. Figure 2 provides a visual summary of the following studies.

### 3.1 Finerenone in cardiovascular diseases

An excessive activation of the RAAS system represents one of the hallmark pathophysiological features of heart failure (46). In this case, aldosterone binds with MR, leading to cardiac fibrosis and subsequent impaired cardiac functions (42, 47, 48). However, finerenone may exert cardiac anti-fibrotic effects, which are distinct from those of other MRAs, by selectively modulating the recruitment of MR co-factors. For instance, after inducing short-term cardiac fibrosis in a mouse model using isoproterenol, Jana Grune et al. demonstrated that both eplerenone and finerenone could significantly inhibit an isoproterenol-induced increase in the left ventricular mass, but only the latter could effectively prevent cardiac fibrosis and macrophage infiltration, while markedly suppressing the expression of tenascin-X (TNX), the gene associated with cardiac fibrosis. Interestingly, these effects were not observed with eplerenone, thus highlighting finerenone's greater potency and reverse remodeling activity (11). Further analysis in the FIDELIO-DKD trial revealed that patients treated with finerenone experienced a significant reduction in the composite cardiovascular outcome compared to those on placebo (49). This outcome included death from cardiovascular causes, non-fatal myocardial infarction, non-fatal stroke, or hospitalization due to heart failure. Specifically, the hazard ratio was 0.86, with a 95% confidence interval ranging from 0.75 to 0.99, and a *P*-value of 0.03, indicating a 14% relative risk reduction. This underscores the cardioprotective effect of finerenone in this patient population. Although these findings were not the primary focus of the trial, they contribute importantly to understanding the broader impact of finerenone and its potential use in mitigating cardiovascular events in patients with T2DM-CKD (20, 50).

Atrial fibrosis, which contributes to structural and electrical remodeling, is recognized as a mechanism underlying atrial fibrillation (AF) (51–54). Preclinical models have demonstrated that finerenone can reduce MR-mediated left atrial enlargement and fibrosis, thereby suggesting a potential mechanism through which this compound may mitigate the onset of arrhythmias such as AF (55). For instance, through a meta-analysis investigating finerenone's impact on atrial fibrillation/flutter (AFF) in a group of CKD patients with type 2 diabetes, Gerasimos Filippatos et al., found that 461 out of 5,674 patients (8.1%) had a history of AFF. However, for the finerenone group (3.2%, 82 cases), the incidence of new-onset AFF was lower compared with the placebo (4.5%, 117 cases), hence suggesting a reduced risk with the use of finerenone (hazard ratio: 0.71; 95% confidence interval: 0.53–0.94; *p* = 0.016). In addition, this compound exerted consistent effects on major renal outcomes (kidney-related death, end-stage kidney disease or sustained eGFR decline of ≥40% in from baseline) and key secondary cardiac outcomes (time to heart failure hospitalization, non-fatal myocardial infarction, cardiovascular death or non-fatal stroke) regardless of the AFF status at baseline (interaction *p*-values 0.16 and 0.85, respectively) (12). However, only a limited number of new AFF events were considered for this secondary analysis from an RCT, thus necessitating caution during interpretation. It is plausible that the analyses overlooked clinically asymptomatic new-onset AFF cases, suggesting the need for larger studies to validate finerenone's effects on AFF.

### 3.2 Finerenone's impact on vascular function

After constructing a myocardial infarction (MI) model by ligating the left coronary artery of MRSMKO mice, Alexandre Gueret et al. found that the progression of post-MI heart failure (HF) was dependent on the MRs in vascular smooth muscle cells (VSMCs). Indeed, the deletion of MR was shown to improve cardiac and coronary endothelial functions and reduce oxidative stress, thereby clarifying the role of VSMC-MR in worsening HF post-MI and identifying it as a potential therapeutic target. In this context, finerenone was found to reduce aldosterone-induced VSMC proliferation and endothelial cell apoptosis in a dose-dependent manner, hence demonstrating improved cardiac and coronary functions possibly as a result of its antagonistic effects on VSMCs’ MR (13). In a different study, Ly Tu et al. discovered the therapeutic potential of finerenone in established pulmonary hypertension (PH) rat models (14). Specifically, they observed increased MR expression in experimental and human Pulmonary Arterial Hypertension (PAH), while in idiopathic PAH patients, finerenone treatment or siRNA-mediated MR silencing reduced the proliferation of pulmonary artery smooth muscle cells (PA-SMCs) (14). Furthermore, after using Sugen/Hypoxia (SuHx) and monocrotaline (MCT) to generate severe PH rat models, finerenone was shown to partially reverse the established PH, induce vascular remodeling and reduce total pulmonary vascular resistance (14). Cellular experiments further revealed that finerenone-based treatments could further reduce the proliferation of PA-SMC as well as downregulate several key MR-regulated genes that control cell cycle and cell proliferation, such as PLK1, MCM5, FZR1, ESPL1, CDC45 and CCND1. Altogether, these findings suggest potential new therapeutic targets for alleviating pulmonary hypertension, but further real-world clinical studies are required for assessing finerenone's efficacy and safety (14).

The effects of finerenone in transgenic rat models of diabetes and hypertension [(mRen-2)27 rats] as well as in a mouse model of oxygen-induced retinopathy (OIR) were also investigated by Jack R. Jerome et al. (15). In this case, both finerenone and the ACE inhibitor perindopril lowered systolic blood pressure in the diabetic rats, reduced retinal gliosis, decreased vascular leakage and reduced the density of retinal microglial cells/macrophages. however, only finerenone reduced the retinal levels of IL-1β, ICAM-1 and VEGF. Similarly, in the OIR mouse model, finerenone reduced vascular leakage, retinal neovascularization as well as the density of retinal microglial cells, while increasing regulatory T cells (Tregs) in the retina, spleen and blood (15). Based on these experimental results, the efficacy of finerenone in diabetic retinopathy could be questioned, and to address this issue, Peter Rossing et al. performed pooled analyses of routine ophthalmic examination data from participants in the FIGARO-DKD and FIDELIO-DKD phase 3 clinical trials in the ReFineDR and DeFineDR studies (56). Of the 244 patients included, 134 received finerenone, and 110 received a placebo. Overall, the results indicated that less patients within the finerenone group developed vision-threatening complications in at least one eye (56), and therefore, it was concluded that finerenone might help delay the progression of non-proliferative diabetic retinopathy, with this potential benefit being independent of baseline HbA1c levels. Finerenone also showed potential benefits in preventing the need for ocular interventions, but even though this compound may represent a potential treatment for diabetic retinopathy, the above findings need to be confirmed through additional randomized studies (56).

### 3.3 Finerenone in renal conditions

In diabetic nephropathy, MR activation is known to induce deleterious glomerular alterations and tubulointerstitial fibrosis (41, 57). However, studies have shown that, by binding to MR, finerenone can prevent pro-fibrotic and pro-inflammatory factors from being transcribed in renal cells such as fibroblasts, endothelial cells, macrophages, mesangial cells and podocytes (58–60). Finerenone's renal protective effects could be the result of its anti-inflammatory, antioxidative and anti-fibrotic properties which delay the progression of diabetic kidney disease (DKD) (10, 61). For instance, in one study, Lan Yao et al. used streptozotocin (STZ) and a high-fat diet to induce type 2 diabetes in C57BL/6J male mice before culturing human renal proximal tubular epithelial cells (HK-2 cells) in media containing high glucose levels. In this case, the results showed that finerenone treatment improved renal morphology, restored mitochondrial ATP content and reduced the serum creatinine levels urinary as well as the albumin-to-creatinine ratio (UACR) of the diabetic mice (16). Furthermore, in addition to reduced apoptosis, mitochondrial fragmentation and oxidative stress, finerenone treatment also restored mitochondrial autophagy via the PI3 K/Akt/eNOS signaling pathway. In fact, diabetic conditions decrease the phosphorylation of PI3 K, Akt, and eNOS, with subsequent finerenone treatment restoring that pathway (16). These findings highlight the potential of finerenone in treating mitochondrial dysfunction in DKD, especially in improving mitochondrial dynamics and autophagy and reducing oxidative stress and apoptosis. Similarly, in a different study, Lionel Lattenist et al. reported that finerenone prevented the development of CKD as well as ischemia/reperfusion (IR)-induced acute kidney injury in Wistar rat models by affecting ET-B receptors and preventing oxidative stress (17).

Beside basic research, the findings of clinical trials have also suggested that, in patients with advanced DKD, finerenone can reduce cardiovascular mortality, a decline in eGFR and the occurrence of end-stage renal diseases (20, 21, 62). Consequently, for patients with T2DM-related CKD who have persistent albuminuria despite standard treatments, the ADA recommends finerenone for slowing kidney disease progression (63). Notably, SGLT2 inhibitors (SGLT2i) are recognized for improving cardiovascular and renal outcomes in both diabetic and non-diabetic patients, with the primary renal mechanism being the inhibition of glucose reabsorption. This inhibited process subsequently induces glycosuria and natriuresis, leading to normalized tubuloglomerular feedback (64, 65). SGLT2i treatment was given to 6.7% of the patients in the FIDELITY analysis, but the outcomes did not differ between these patients and those not on SGLT2i, hence indicating that combining finerenone with SGLT2i may have additive renal protective effects (10). Moreover, DKD patients undergoing treatment commonly discontinue certain medications due to hyperkalemia, and in this case, SGLT2i can lower risks of this adverse event through its moderate potassium-lowering effect (10, 66, 67). This was supported by the FIDELIO-DKD study where post-hoc analysis indicated a 55% lower risk of hyperkalemia by combining finerenone with SGLT2i (HR, 0.45; 95% CI, 0.66–0.87; *P* < 0.0001) in comparison with the non- SGLT2i group (10). However, caution is required when interpreting those results since, in that study, SGLT2i was given to only 4.6% of the participants (68). Nevertheless, in the long run, considering a combination of SGLT2i and finerenone for DKD patients may yield cumulative benefits (69, 70).

### 3.4 Effects of finerenone on metabolic functions

Vincenzo Marzolla et al. induced obesity in a mouse model using a high-fat diet (HFD) to explore the metabolic effects of finerenone, with the results showing the activation of the AMPK-ATGL-UCP-1 signaling pathway as well as improved glucose tolerance under HFD conditions following treatment with finerenone (18). This was particularly noticeable in terms of increased functionality of brown adipose tissues (BATs) which use excess energy through thermogenesis. In this case, the underlying mechanism of finerenone involved increased AMPK activation which, in turn, stimulated ATGL activation and led to increased UCP-1 expression associated with enhanced BAT thermogenic function (18). Therefore, this study suggested a potential pharmacological approach for treating metabolic diseases related to adipose tissue dysfunction using finerenone although such treatment may also provide additional cardiovascular benefits in patients with metabolic syndromes.

Notably, in 2023, through a scientific statement on Cardiovascular-Kidney-Metabolic (CKM) syndrome, the AHA proposed a CKM staging system for the early identification of CKM syndromes in order to prevent the development of clinical cardiovascular disease and renal failure (19, 71). This staging method emphasizes the complex interactions between metabolic risk factors, CKD and cardiovascular disease by providing a comprehensive framework to assess and intervene in these interrelated conditions (19). This staging system is divided into the following stages: Stage 0: no CKM health risk factors; Stage 1: excess and/or dysfunctional adipose tissues; Stage 2: metabolic risk factors and CKD; Stage 3: subclinical cardiovascular diseases in CKM; and Stage 4: clinical cardiovascular diseases in CKM (19). The AHA discussion also revolved around the potential benefits of using finerenone at each stage of the CKM syndrome, especially at Stage 3 CKM (patients with subclinical cardiovascular diseases or heart failure and CKM risk factors or equivalent risks) where the drug help to prevent the progression of renal and cardiovascular diseases (19).

Preclinical models as well as clinical trials have largely established the beneficial effects of finerenone, with this novel non-steroidal MR antagonist improving cardiac remodeling, reducing the risk of atrial fibrillation, decreasing the UACR, improving mitochondrial dysfunction and regulating metabolism. Additionally, potential combinations with existing treatments, such as SGLT2 inhibitors (SGLT2i), offer new insights into finerenone's practical application in the integrated management of cardio-renal-metabolic syndrome. However, despite the extensive therapeutic potential of finerenone, its specific mechanisms of action and clinical efficacy at different stages of disease still need to be clarified through additional research in view of establishing its therapeutic role for renal and cardiovascular diseases.

In the previous sections, the pharmacological effects of finerenone and relevant basic research were discussed. Specifically, finerenone has shown positive pharmacological actions in terms of cardiac anti-fibrotic and metabolic effects, in protecting vascular function and in slowing DKD's progression. Basic research can serve as the starting point for clinical practice, and subsequent research should now focus on assessing finerenone's performance in clinical settings. So far, a number of key clinical studies have offered valuable data on the compound's safety and efficacy, and these will be reviewed in subsequent sections to assess the potential applications of finerenone in real-world medical scenarios.

## 4 Major clinical studies on finerenone

The current review provides an overview of the major clinical trials involving finerenone so far, and these include ARTS, ARTS-HF, ARTS-DN, FIDELITY, FIGARO-DKD and FIDELIO-DKD, along with their respective sub-studies. Table 2 offers a detailed summary of the clinical studies involved below.

**Table 2:** Pivotal trials on finerenone.

Author/year	Acronym	Drugs compared/dose protocol	Total participants	Population	Primary endpoints	Findings	Follow-up duration
Pitt et al., (23)	ARTS	Finerenone 2.5–10 mg daily or Spironolactone 25–50 mg daily	392	HFrEF (LVEF ≤ 40%) +eGFR 30–60 ml/min	Part A The effects on serum potassium concentration, eGFR, and albuminuria were assessed Part B The change in serum potassium concentration after treatment	1. Compared with spironolactone, BAY 94-8862 resulted in a significantly smaller increase in serum potassium concentration (0.04–0.30 mmol/L vs 0.45 mmol/L, *P* < 0.0001–0.0107). 2. The incidence of hyperkalemia was lower in the BAY 94-8862 group (5.3%) compared to the spironolactone group (12.7%, *P* = 0.048). 3. BAY 94-8862 reduced levels of BNP, NT-ProBNP, and albuminuria, at least as much as spironolactone. 4. Adverse events associated with BAY 94-8862 were infrequent and mostly mild.	4 wks
Filippatos et al., (24)	ARTS-HF (NCT01807221)	Finerenone 2.5–20 mg daily or eplerenone 25–50 mg daily	1,066	HFrEF (LVEF ≤ 40%) + DM and/or CKD	Percentage of patients with a >30% decrease in NT-ProBNP from baseline to day 90	1. In the 10 to 20 mg orally once daily, there was a nominal benefit (HR: 0.56; 95% CI, 0.35–0.90; *P* = 0.02). 2. Hyperkalemia (>5.6%): 4.3% in finerenone group.	3 mo
Bakris et al., (25)	ARTS-DN (NCT1874431)	Finerenone 1.25–20 mg daily	823	DM + eGFR > 30 ml/min +albuminuria ≥ 30 mg/g	Urine albumin creatinine ratio at day 90 vs. at baseline	1. Urine albumin creatinine ratio reduction: ○ Finerenone: 7.5 mg/day, (0.79; 90% CI 0.68–0.91; *P* = .004); for 10 mg/day (0.76; 90% CI 0.65–0.88; *P* = .001); for 15 mg/day,(0.67; 90% CI 0.58–0.77;*P* < .001); for 20 mg/day (0.62; 90% CI 0.54–0.72; *P* < .001) 2. Hyperkalemia: incidences in the finerenone 7.5, 15, and 20-mg/day groups were 2.1%, 3.2%, and 1.7%, respectively.	3 mo
Pitt et al., (21)	FIGARO-DKD (NCT02545049)	Finerenone 10 or 20 mg daily Matching placebo daily	7,437	DM + eGFR > 25–90 ml/min and albuminuria 30–300 mg/g or eGFR > 60 ml/min and albuminuria 300–5,000 mg/g	Number of participants with the first occurrence of the primary CV composite outcome, CV death, non-fatal MI, non-fatal stroke, or HHF	1. Finerenone therapy improved cardiovascular outcomes. Finerenone vs. Placebo: 12.4% vs. 14.2% (HR, 0.87; 95% CI, 0.76 −0.98; *P* = 0.03) 2. Composite endpoints for renal outcomes Finerenone vs. Placebo 9.5% vs. 10.8% (HR 0.87; 95% CI, 0.76–1.01) 3.The incidence of hyperkalemia was higher. Finerenone vs. Placebo 10.8% VS 5.3%	3.4 yrs
Bakris et al., (20)	FIDELIO-DKD (NCT02540993)	Finerenone 10 or 20 mg daily Matching placebo daily	5,734	DM + eGFR 25–60 ml/min and albuminuria 30–300 mg/g or eGFR 25–75 ml/min and albuminuria 300–5,000 mg/g	Count of participants and time from randomization to the first occurrence of the primary renal composite outcome, onset of kidney failure, a sustained decrease of eGFR ≥40% from baseline over at least 4 weeks, or renal death were evaluated	1. Finerenone treatment resulted in lower risks of CKD progression than placebo Finerenone v. Placebo 17.8% vs. 21.1%, (HR0.82, 95% CI 0.73–0.93, *P* = 0.001) 2.Cardiovascular events Finerenone vs. Placebo 13.0% vs. 14.8%, (HR, 0.86; 95% CI, 0.75–0.99; *P* = 0.03) 3. Incidence of discontinuation of the trial protocol due to hyperkalemia (2.3% vs. 0.9%)	2.6 yrs
Filippatos et al., (49)	FIDELITY substudy: FIDELIO + FIGARO	Finerenone 10 or 20 mg daily Matching placebo daily	13,026	DM + eGFR > 25–90 ml/min and albuminuria 30–300 mg/g or eGFR > 25 ml/min and albuminuria 300–5,000 mg/g	The effects of finerenone on cardiovascular and kidney outcomes in patients with T2DM and CKD	Finerenone reduced the risk of clinically important cardiovascular and kidney outcomes vs. placebo across the spectrum of CKD in patients with type 2 diabetes 1. Cardiovascular outcomes Finerenone vs. Placebo 12.7% vs. 14.4%, (HR0.86, 95% CI 0.78–0.95, *P* = 0.0018) 2.Kidney outcomes Finerenone vs. Placebo 5.5% vs. 7.1%, (HR0.77, 95% CI 0.67–0.88, *P* = 0.0002) Incidence of discontinuation of the trial protocol due to hyperkalemia (1.7% vs 0.6%)	3 yrs
None	FINEARETS-HF (NCT04435626)	Finerenone 10–40 mg daily Matching placebo daily	6,001	NYHA class II-IV and LVEF ≥ 40%	Number of cardiovascular deaths and heart failure events	Finerenone reduced the composite endpoint of cardiovascular death and total heart failure events (first and recurrent) compared to placebo.	42 mo

A large, randomized, double-blind clinical trial involving heart failure patients presenting reduced ejection fraction (HFrEF; NYHA class II-III) as well as mild to moderate CKD was performed by Pitt et al. to evaluate finerenone's tolerability and safety. The study was divided into two parts: Part A, which included 65 patients, assessed the safety of different doses of finerenone in patients with mild CKD which was defined as an eGFR of 60–90 ml/min/1.73 m^2^); On the other hand, Part B, involving 392 patients, of whom 56 had previously received MRA therapy, compared finerenone's safety to that of spironolactone or placebo in patients with moderate CKD, with the latter defined as an eGFR of 30–60 ml/min/1.73 m^2^). Overall, it was noted that the incidence of hyperkalemia and elevated serum potassium levels was lower for finerenone (2.5–10 mg, once or twice daily) in comparison with spironolactone (25–50 mg/day). Additionally, finerenone showed a dose-dependent lowering of BNP and NT-ProBNP levels alongside improved UACR as opposed to spironolactone. The results demonstrate the potential advantages of finerenone in cardio-renal protection, hence offering a new therapeutic option for heart failure and CKD (23). Although this study did not use MACE events as hard endpoints to evaluate efficacy, the improved BNP levels and the UACR value highlight finerenone's potential for cardio-renal protection. Its ability to maintain electrolyte balance further underscores its safety and tolerability in the treatment of such conditions.

For the ARTS-HF study, finerenone's efficacy and safety was assessed in patients with worsening chronic heart failure alongside concomitant diabetes and/or CKD. This randomized, double-blind and active-controlled phase IIb study by Filippatos et al. involved 1,060 patients with HFrEF and it compared finerenone to eplerenone, with the endpoints being changes in health-related quality of life and the impact of finerenone on composite clinical endpoints (72). The finerenone group received doses that ranged from 2.5 to 15 mg daily as per a titration scheme, with the doses subsequently adjusted after day 30. On the other hand, 25 mg of drug was given every other day to patients within the eplerenone group, with the dose then increasing to once daily before reaching 50 mg daily by day 60. In this case, the percentage of individuals with more than 30% reduction in plasma NT-proBNP levels after 90 days was determined as the primary endpoint. Overall, the two drugs did not differ significantly in their potential to reduce NT-ProBNP (*P* = 0.42–0.88), although statistically significant improvement was still observed for the 10-mg finerenone group after dose escalation (HR 0.56; 95% CI 0.35–0.90, *P* = 0.02). Regarding safety, incidences of hyperkalemia were balanced between the groups, with the 4.3% of cases within the finerenone group being comparable to that of the eplerenone group (24). This study primarily compared the safety and tolerability of finerenone against the second-generation MRA antagonist eplerenone at different dosages. The observed outcomes were similar to those in the ARTS study, except that the primary efficacy endpoint was only based on NT-proBNP reduction rather than hard endpoints such as mortality or hospitalization rates in order to provide more clinical relevance. However, the potential clinical benefits of finerenone on exploratory endpoints and quality of life (QoL) assessments also provide a groundwork for future studies focusing on relevant hard endpoint outcomes.

Finerenone's safety and efficacy in DKD patients were assessed in the multicenter, randomized and double-blind ARTS-DN clinical study by Kabris et al. This trial, involving a controlled placebo and parallel groups included 821 patients who had received ACEi/ARB treatment and who repesented high or very high proteinuria. The treatment period was 90 days, with the dosage being in the range of 1.25–25 mg once daily. In this case, a change in the UACR value was determined as the primary endpoint, while eGFR values and changes in the serum level of potassium were taken as the safety endpoints. Overall, the findings suggested that, at day 90, finerenone could significantly reduce UACR at doses of 7.5, 10, 15 and 20 mg compared with baseline (*P*-values were 0.004, 0.001, <0.001, and <0.001, respectively). Regarding safety, hyperkalemia's occurrence within the finerenone and placebo groups were not significantly different, with the dosage groups being also statistically similar in terms of the secondary endpoints (adverse events, serious adverse events and a >30% reduction in eGFR). Therefore, finerenone displayed a dose-dependent improvement in UACR in the treatment of DKD (25, 73). This research, focused on DKD patients with proteinuria, not only extended the findings of the ARTS study on UACR improvement, but also provided evidence that different doses of finerenone were safe and effective for reducing albuminuria in such patients, thereby guiding future clinical practice and research directions. However, the ARTS-DN study had a short treatment duration and follow-up period, with kidney outcome endpoints being also absent. As a result, it may be insufficient to fully assess finerenone's long-term impact on the progression of CKD.

The FIDELIO-DKD trial was a multicenter, randomized and double-blind phase III study that included a controlled placebo alongside parallel groups. Performed by Bakris et al., this event-driven clinical trial involved 5,734 CKD and T2DM patients who received background ACEI or ARB therapy. These patients were then assigned to two groups for CKD: the first one included those having a history of diabetic retinopathy, persistent moderate albuminuria (UACR 30 to <300 mg/g) and an eGFR of 25 to <60 ml/min/1.73 m^2^. The other group included patients who presented persistent severe albuminuria (UACR 300 to 5,000 mg/g) along with an eGFR of 25 to <75 ml/min/1.73 m^2^. During the follow-up period (median of 2.6 years), a significantly lower (17.8% vs. 21.1%; HR, 0.82; 95% CI, 0.73 to 0.93; *P* = 0.001) risk of the primary endpoint (renal-related death, kidney failure or sustained eGFR decline of ≥40% from baseline) alongside significantly lower (13.0% vs. 14.8%; HR, 0.86; 95% CI, 0.75–0.99; *P* = 0.03) key secondary endpoints (composite of hospitalization for heart failure, non-fatal myocardial infarction, cardiovascular death or non-fatal stroke) were noted for the finerenone group compared with placebo. Regarding safety outcomes, the two groups were similar in their overall frequency of adverse events, although more patients from the finerenone group (2.3%) discontinued the trial discontinuation as a result of hyperkalemia as opposed to the placebo group (0.9%). Interestingly, despite all patients receiving renin-angiotensin system (RAS) blockade therapy, the finerenone group still demonstrated additional renal and cardiovascular protective effects. In summary, in T2DM and CKD patients, finerenone treatment reduced the risks of cardiovascular events and CKD progression in comparison with placebo, with the overall incidence of adverse events being also balanced between the two groups (20).

Based on the previous study, B. Pitt et al. conducted the FIGARO-DKD trial, a multicenter, randomized and double-blind phase III clinical study involving a controlled placebo group along with parallel groups. The aim of that event-driven trial was to compare finerenone's safety and efficacy against placebo in DKD patients receiving standard medical therapy. For this purpose, the study population was first assigned to two categories: one group involved patients at CKD stages 2–4 (eGFR 25–60 ml/min/1.73 m^2^) with a UACR of 30–300 mg/g and persistent moderately increased albuminuria, while the other one included patients at CKD stages 1–2 (eGFR ≥60 ml/min/1.73 m^2^).with a UACR of 300–5,000 mg/g and persistent severely increased albuminuria. However, the study excluded patients with symptomatic HFrEF as well as those who were highly represented in the FIDELIO-DKD trial (eGFR 25 to <60 ml/min/1.73 m^2^ and UACR 300 to 5,000 mg/g). Overall, 7,437 patients were randomized, with the primary outcome being a composite of hospitalization for heart failure, non-fatal myocardial infarction, cardiovascular death or non-fatal stroke. In addition, the secondary outcomes included renal-related death, kidney failure or a sustained eGFR decrease of ≥40% from baseline. Finally, safety was assessed based on reported adverse events. In this case, a significantly lower primary composite outcome [458 of 3,686 patients (12.4%)] was noted for the finerenone group in comparison with placebo [519 of 3,666 patients (14.2%)] (HR 0.87, 95% CI 0.76–0.98, *P* = 0.03), along with less hospitalization due to heart failure (117 patients [3.2%] vs. 163 patients [4.4%], HR 0.71, 95% CI 0.56–0.90, *P* = 0.03). Furthermore, secondary outcome events were noted in 350 (9.5%) and 395 (10.8%) patients for the finerenone and placebo groups, respectively (HR 0.87, 95% CI 0.76–1.01). However, these two groups did not differ in their overall frequency of adverse events, although for the finerenone group, higher hyperkalemia resulted in the trial being discontinued for more patients (1.2% vs. 0.4%). In summary, patients with T2DM and CKD exhibited improved cardiovascular outcomes after finerenone treatment, especially through less hospitalizations for heart failure (21, 74–76).

Based on the FIGARO and FIDELIO studies, Agarwal et al. conducted a pre-specified pooled analysis (FIDELITY study) at the individual patient level to reliably estimate finerenone's efficacy and safety as opposed to placebo in a broad range of CKD patients. Specifically, the study included T2DM + CKD patients with an eGFR of ≥25 ml/min/1.73 m^2^, while excluding those with symptomatic HFrEF (NYHA class II-IV). During the follow-up period (median of 3.0 years), 14% less cardiovascular composite outcomes (hospitalization for heart failure, non-fatal myocardial infarction, cardiovascular death or non-fatal stroke) (HR, 0.86; 95% CI, 0.78–0.95; *P* = 0.0018) as well as 23% less renal composite outcomes (renal death, kidney failure or a sustained eGFR decline of ≥57% from baseline) (HR, 0.77; 95% CI, 0.67–0.88; *P* = 0.0002) were noted for the finerenone group in comparison with placebo. However, 1.7% of patients within the finerenone group (0.6% for the placebo group) also had to discontinue treatment due to hyperkalemia which was the most common adverse event associated with the drug, although clinically significant hyperkalemia-related adverse events remained infrequent (22, 77). The FIGARO-DKD and FIDELIO-DKD studies, conducted sequentially, complement each other in revealing finerenone's safety and efficacy in treating different stages of T2DM-CKD. Specifically, the FIDELIO-DKD study focused on patients presenting severe albuminuria alongside stage 3 or 4 CKD, thus highlighting the compound's protective effects on the kidneys (78, 79). On the other hand, patients with a broader range of CKD stages were included in the FIGARO-DKD study, with the latter also laying emphasis on finerenone's cardiovascular protective effects (78, 79). Additionally, the FIDELITY analysis further reinforced the reduced risk of kidney disease progression and cardiovascular events in T2DM + CKD patients who had been treated with finerenone. Collectively, these studies support the therapeutic concept of cardio-renal integrated management, while confirming the positive impact of finerenone on clinical outcomes. However, current research has, so far, been largely focused on T2DM-CKD patients. Consequently, future studies need to consider non-diabetic CKD patients in order to encompass a broader patient population. Additionally, more detailed analyses of heart failure patients are required to ensure comprehensive and relevant research results that can better guide clinical practice.

A comprehensive examination of the progress in finerenone research provides strong evidence regarding its potential in the treatment of cardiorenal diseases (80). Indeed, in-depth basic research has revealed the compound's multifaceted mechanisms which range from reduced cardiac fibrosis and optimized vascular endothelial functions to the intricate regulation of metabolic pathways. Furthermore, landmark clinical studies such as FIDELIO-DKD and FIGARO-DKD have further validated its protective role in T2DM-CKD patients. However, despite its potential, finerenone's clinical application remains challenging. In particular, its future prospects for broader patient groups and diversified therapeutic indications need to be comprehensively assessed while taking into account the potential adverse effects for long-term treatment. Nevertheless, despite challenges, it is expected that future research to address these areas will provide valuable opportunities not only to expand finerenone's therapeutic boundaries but also to deepen medical understanding on its suitability in clinical treatment.

## 5 Opportunities and challenges of finerenone

Although many large clinical studies have confirmed finerenone's safety and efficacy in T2DM-CKD patients, data are still lacking regarding its efficacy in non-diabetic CKD (ndCKD) patients. However, a real-world study involving 43 ndCKD patients in China yielded conclusions that were relevant to this issue. Indeed, in that study, membranous nephropathy was confirmed in 46.5% of the patients, but when finerenone was added to their ongoing standard CKD therapy, the patients’ 24-h urinary total protein was significantly reduced by 60.86% (IQR, 35.625%-87.062%), with a median reduction of 2.76 g (IQR, 0.2015–2.609, *p* < 0.01). Furthermore, throughout the follow-up period, the eGFR and serum potassium levels remained stable, thus confirming the compound's efficacy and safety in ndCKD patients. However, to validate these results, large prospective studies are still required (81). Currently, phase III of the FIND-CKD study (NCT05047263) targeting the treatment of CKD patients without T2DM has been initiated (82). That study aims to assess whether, on top of standard treatment and well-controlled risk factors, the use of finerenone can provide additional renal protection while delaying kidney disease progression (83).

As a novel non-steroidal selective MRA, finerenone has demonstrated potential in reducing the risk of cardiovascular and renal events associated with T2DM-CKD (84). However, its efficacy in patients with CKD related to type 1 diabetes (T1D) is yet to be established. The FINE-ONE trial, a phase III, randomized, placebo-controlled, double-blind study, aims to assess the efficacy and safety of finerenone in T1D and CKD patients. Approximately 220 eligible individuals will be randomized to receive finerenone or placebo, with the primary endpoint being the relative change in UACR over six months (85). Preliminary animal model studies suggest potential renoprotective and cardioprotective effects of finerenone in T1D, but its application in human T1D patients awaits the results of the FINE-ONE trial (86). The study's findings may position finerenone as the first registered treatment for T1D-related CKD in nearly three decades.

As MR antagonists, steroidal MRAs, such as spironolactone, are commonly used to treat primary aldosteronism (87, 88). However, insufficient data and clinical evidence are available regarding the application of finerenone for the same purpose. Currently, two investigator-initiated cohort studies (NCT05924620 and NCT05814770, with sample sizes of 60 and 96, respectively) involving this condition are being undertaken. In these studies, the control group receives spironolactone plus standard of care (SOC), while the experimental one uses finerenone plus SOC. So far, so related outcomes have been observed, but it is expected that the results will further expand finerenone's application scope if the data support its efficacy in the treatment of primary aldosteronism (89, 90).

In the aforementioned clinical studies, although finerenone demonstrated relevant advantages; the occurrence of hyperkalemia remains a potential risk, and hence, regular monitoring of potassium levels and appropriate adjustments to the medication regimen is required (68, 91). Beyond this common adverse effect, the association between finerenone and cancer risks also warrants attention. In this context, a meta-analysis of four RCTs involving 14,875 participants assessed the risk of tumors in T2DM-CKD patients who had been treated with finerenone. Although the analysis found no association between finerenone and overall tumor risk (including malignant and benign tumors), it still suggested a potential increase in the risk of urinary tract malignancies (Peto OR = 1.69; 95% CI, 1.07–2.67) (92). However, that study had a limited sample size and lacked follow-up duration while having a low to moderate risk of bias. Therefore, the above conclusions need to be validated through additional well-designed studies involving broader populations.

In the FIDELITY study, Finerenone showed potential in reducing sudden cardiac death risk for patients with T2DM and CKD. Its mechanisms might involve boosting the protective effects of ACE I/ARBs against cardiac remodeling and neutralizing aldosterone's proarrhythmic effects (22). Baseline conditions like atrial fibrillation or heart failure could affect sudden cardiac death rates, and Finerenone may modulate these risks (22, 93). By keeping serum potassium levels normal, it could also decrease arrhythmia risks, key to preventing sudden cardiac death (22, 94). The lower sudden cardiac death incidence in the Finerenone group supports its potential to enhance cardiovascular outcomes (95). Confirmation of these benefits could redefine the use of antiarrhythmic drugs and ICDs.

FINEARTS-HF, a study involving symptomatic heart failure patients (LVEF ≥40% and NYHA class II-IV), is being performed to assess finerenone's safety and efficacy in reducing the occurrence of heart failure exacerbations and subsequent mortality. The trial, which includes 6,001 participants, compares 10, 20 and 40 mg of finerenone (once daily) against placebo, based on eGFR, for a treatment period of up to 42 months. A composite of total heart failure events (first and recurrent) and cardiovascular death was taken as the primary endpoint, while the secondary ones included total HF events (first and recurrent), change in KCCQ-TSS from baseline to months 6, 9 and 12, change in NYHA class from baseline to month 12, a renal composite endpoint (sustained eGFR decline to <15 ml/min/1.73 m^2^, sustained decline in eGFR of ≥50% from baseline for at least 4 weeks, initiation of dialysis or kidney transplantation) and all-cause mortality. Compared to previous HFmrEF/HFpEF trials, participants in FINEARTS-HF were more likely to have a history of heart failure hospitalization within 6 months, more severe symptoms and functional limitations, and a higher proportion of SGLT2 inhibitors and ARNI use. The study results demonstrated that finerenone significantly reduced the composite endpoint of cardiovascular death and total heart failure events (first and recurrent) compared to placebo, with a favorable safety profile (96–98).

Based on evidence-based confirmation of clear cardiorenal benefits, finerenone has been incorporated into multiple authoritative guidelines. Initially, the CKM guidelines mentioned above suggest that the use of finerenone in stage 3 CKM may play a significant role in preventing the progression of cardiovascular and renal diseases (19). Subsequently, the 2024 American Diabetes Association (ADA) “Standards of Care in Diabetes” (Level A recommendation, 4 endorsements) advise: 1. For patients with T2DM-CKD and proteinuria treated with the maximum tolerated dose of ACEi/ARB, the concomitant use of finerenone is recommended to improve cardiovascular outcomes and reduce the risk of CKD (A); 2. In T2DM-CKD patients, consider the use of SGLT-2i (eGFR ≥ 20 ml/min/1.73 m^2^), GLP-1RA, or non-steroidal mineralocorticoid receptor antagonists (eGFR ≥ 25 ml/min/1.73 m^2^) (A); 3. For CKD patients with proteinuria who are at increased risk of cardiovascular events or CKD progression, non-steroidal mineralocorticoid receptor antagonists proven effective in clinical trials are recommended to delay CKD progression and reduce the risk of cardiovascular events, with concurrent monitoring of serum potassium (A); 4. For patients with T2DM and diabetic nephropathy, the use of finerenone is recommended to reduce the risk of hospitalization due to heart failure (A) (99). Finally, “Multidisciplinary expert consensus for clinical application of mineralocorticoid receptor antagonists in China” published in 2022, which recommends the use of finerenone to treat T2DM-CKD, to reduce urinary protein, delay the continuous decline of renal function, and lower the risk of end-stage renal disease, cardiovascular death, non-fatal myocardial infarction, and hospitalization due to heart failure, with spironolactone and eplerenone being considered at discretion. For the application of finerenone in HFrEF, HFmrEF, and HFpEF populations, it is also considered at discretion (100). Overall, there is a lack of studies on spironolactone and eplerenone for improving cardiovascular and renal event endpoints in T2DM-CKD, while finerenone has clear evidence. These major guidelines provide important guidance for the rational use of finerenone in clinical practice, and clinicians should strictly follow the guidelines’ recommended indications and application criteria to benefit more patients.

## 6 Conclusion

RAAS's activation is crucial for the pathophysiology of renal and cardiovascular diseases. Mineralocorticoid receptors are largely present in blood vessels, the kidneys and the heart, and when excessively activated, they induce the production of reactive oxygen species which results in fibrotic processes as well as inflammatory responses, These subsequently lead to conditions, such as myocardial hypertrophy, glomerular hypertrophy and glomerulosclerosis, that ultimately contribute to adverse cardiovascular or renal outcomes. Therefore, blocking MR overactivation is crucial to prevent the above complications. In this context, unlike the traditional MRAs, the distribution of the novel non-steroidal MR antagonist finerenone is more balanced between the kidneys and the heart. Furthermore, its antifibrotic and anti-inflammatory properties as well as its ability to improve endothelial functions, optimize metabolism, reduce oxidative stress and positively impact pulmonary arterial pressure are well established. Finally, as far as finerenone's potential is concerned, recent randomized controlled trials (RCTs) have revealed its clinical efficacy, thereby highlighting its wide scope for future research. It is expected that additional evidence-based medical data will further support finerenone's efficacy in improving the prognosis of cardiovascular disease patients, thus providing a strong scientific basis for its application in clinical treatment.
